# Routes for GMR-Sensor Design in Non-Destructive Testing

**DOI:** 10.3390/s120912169

**Published:** 2012-09-05

**Authors:** Matthias Pelkner, Andreas Neubauer, Verena Reimund, Marc Kreutzbruck, Andreas Schütze

**Affiliations:** 1 BAM Federal Institute for Materials Research and Testing, Berlin 12200, Germany; E-Mails: andreas.neubauer@bam.de (A.N.); verena.reimund@bam.de (V.R.); marc.kreutzbruck@bam.de (M.K.); 2 Laboratory for Measurement Technology, Saarland University, Saarbrücken 66123, Germany; E-Mail: schuetze@lmt.uni-saarland.de

**Keywords:** giant magneto resistance, non-destructive testing, magnetic flux leakage, sensor array

## Abstract

GMR sensors are widely used in many industrial segments such as information technology, automotive, automation and production, and safety applications. Each area requires an adaption of the sensor arrangement in terms of size adaption and alignment with respect to the field source involved. This paper deals with an analysis of geometric sensor parameters and the arrangement of GMR sensors providing a design roadmap for non-destructive testing (NDT) applications. For this purpose we use an analytical model simulating the magnetic flux leakage (MFL) distribution of surface breaking defects and investigate the flux leakage signal as a function of various sensor parameters. Our calculations show both the influence of sensor length and height and that when detecting the magnetic flux leakage of μm sized defects a gradiometer base line of 250 μm leads to a signal strength loss of less than 10% in comparison with a magnetometer response. To validate the simulation results we finally performed measurements with a GMR magnetometer sensor on a test plate with artificial μm-range cracks. The differences between simulation and measurement are below 6%. We report on the routes for a GMR gradiometer design as a basis for the fabrication of NDT-adapted sensor arrays. The results are also helpful for the use of GMR in other application when it comes to measure positions, lengths, angles or electrical currents.

## 1. Introduction

Since its discovery in 1988 [1,2] the giant magneto resistance (GMR) effect has been intensively investigated. This led, e.g., to a major boost in computer hard drive technology by means of smaller read heads resulting in an enhanced bit density. Generally, nowadays the GMR is of interest for many other applications concerning the determination of magnetic fields due to its resistance change of 10–20% at room temperature, its remarkable field sensitivity and detection limit down to the pT-range [3–5]. GMR sensors are prevalent in many different measurement applications such as proximity, position, rotational speed, angle, and electrical current. They can be easily miniaturized and their low power consumption is a further promising feature. Even though they are still relatively costly compared to their semiconducting counterpart—the Hall sensor—the GMR continuously claims further segments in the market, such as automation and production processes, automotive, cell phones, medical application and safety inspection. The latter includes all kind of electromagnetic testing methods to test the integrity of a component. Here, the trend is driven by growing safety requirements in which industries call for reliable non-destructive testing (NDT) methods, especially when it comes to detect small surface breaking defects in the μm-range. In recent years, GMR sensors have been intensively used as magnetic field sensors in magnetic flux leakage (MFL) [6,7] and in eddy current (EC) testing [8–12]. Due to their main promising properties-the high field sensitivity and the high spatial resolution-also small defects can be quantitatively detected paving the way for automation of the testing process.

**Figure 1.**
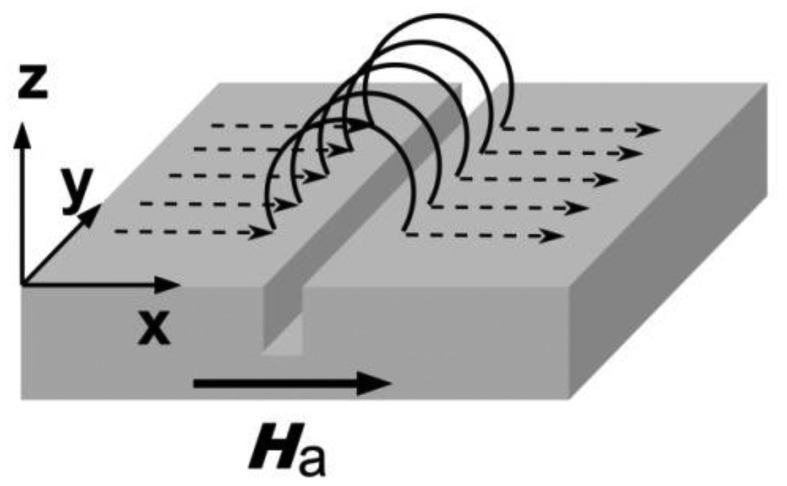
Scheme of the MFL of a crack. Surface breaking magnetic field lines in the presence of discontinuities.

In MFL-testing, a magnetized component shows a flux leakage at defect positions where the permeability significantly differs from the bulk magnetic properties (see Figure 1). In case of conventional MFL, using magnetic particles to visualize the stray field, no quantitative estimation of the leakage field is achievable. In those cases the estimation of the crack depth turns out to be non-feasible and alternative techniques like an electrical potential probe have to be used. In contrast, the quantitative knowledge of the MFL-distribution can be obtained immediately using adapted GMR sensor arrays.

For a reliable defect evaluation a precise characterization of the MFL is necessary [13]. The amount of information can be increased by enhancing the signal-to-noise ratio (SNR) and the spatial resolution. However, most commercially available GMR sensors are not designed for NDT applications. They are integrated in encapsulations, resulting in a large, detrimental distance between their active layers and the surface of the component to be tested. This proves to be disadvantageous, since the amplitude of the MFL decreases strongly with increasing sensor-to-surface distance; it is approximately inversely proportional to the distance squared for larger distances. As a direct consequence the SNR decreases with increasing distance resulting in noisier signals. As a further result of increased sensor-to-defect separation one obtains more or less blurred field signals showing broader field distributions which makes an interpretation more difficult. Therefore, a distance- and size-adapted GMR design is desirable for NDT applications in order to increase the reliability of the detection of small defects and also the reconstruction of cracks.

In NDT industries measurement time is a key feature. Therefore, using a single small sized GMR sensor element to scan an object in a fine grid is in most cases not practicable. Alternatively, a sensor array housing several tens of simultaneously operating elements is more reasonable. In this paper we present a parameter study investigating the optimal arrangement, orientation and dimensions of active GMR layers. The analysis is based on an analytical model calculating the stray field of surface breaking defects introduced by Shcherbinin *et al.* [14,15] and improved by Foerster [16]. First, we deal with the different gradiometric arrangements of the sensing elements, where two magnetometer signals are subtracted from each other in order to reduce background clutter. Next, concentrating on a normal gradiometric sensor arrangement, we present a parameter study of the dimension and the layout of the active sensing layers. The results and consequences are discussed in the following. Finally, we present a comparison of simulated results with measurements carried out using a GMR magnetometer.

## 2. Magnetic Flux Leakage Signals

MFL signals (a scheme of a MFL-scenario is shown in Figure 1) occur in the presence of surface breaking defects in magnetized ferromagnetic materials. The strength of the MFL signal depends on the magnetization of the component, the permeability, and on the geometry of the defect, in which the defect depth contributes most and the crack opening (gap in *x*-direction in Figure 1) has only a negligible influence on the signal strength. Furthermore, the sensor-to-surface distance plays an essential role due to the 1/*r*^2^-dependence of the magnetic stray field.

Both, analytical and finite-element methods (FEM) can be used to quantitatively calculate the magnetic stray fields. Analytical approaches allow a fast way of calculation and achieve for simple geometries, as it is the case in this study, similar accuracies as FEM, which generally comes along with high computational costs. In 1966 Zatsepin and Shcherbinin [14] introduced an analytical model which evaluates the MFL of a 3D surface-breaking crack with rectangular shape using magnetic dipoles. However, they did not relate the magnitude of the MFL to the magnetic properties of the material and the external applied field. Shcherbinin and Pashagin [15] extended the model to defects with rectangular shape and finite size. A further evaluation of the model was published by Edwards and Palmer [17].

Applying this analytical model [15] we calculated the stray field of a critical crack with the following dimensions: length 500 μm, depth 50 μm, and opening 2 μm. For the applied field we chose *H*_a_ = 100 A/m and a permeability μ_r_ = 1,000. The calculated stray field represents the base for the investigation of the sensor parameters and the arrangement carried out in this work.

## 3. Sensor Type and Arrangement of the Sensing Areas

The GMR active layers can be patterned as simple resistors, half bridges, and Wheatstone bridges. Simple resistors are the smallest configuration and can be arranged with few components. However, the drawback of a simple resistor is its temperature dependence. GMR layers patterned as half bridges and Wheatstone bridges offer a better temperature compensation for the price of a more complicated chip design fitting two or more active layers on a circuit board. In the bridge setup GMR sensors can be used as magnetometers or as gradiometers. In the latter case the active layers are separated by a distance detecting the field gradient.

Magnetometers formed in a Wheatstone bridge generally require some of its active parts being magnetically shielded, whereas these shields are not required for gradiometers. The application of the magnetic shielding is an additional step during the fabrication of the GMR sensors which, if not carried out correctly, easily leads to malfunctioning sensors. Hence, the additional process during the fabrication of magnetometers is—aside from suppressing external noise—an important issue for the use of gradiometric GMR sensors. This becomes especially relevant when aiming for GMR sensor arrays with a large number of sensors since one malfunctioning sensor would decrease the performance of the whole array.

A further drawback of a magnetometer is that it picks up disturbing external magnetic noise. This includes both 50/60 Hz power lines or high current flows in industrial environments and static background fields caused by magnetic materials and, e.g., the geometry of the test object itself. For an appropriate gradiometer with defined base line the level of the external noise can be significantly suppressed depending on the spatial homogeneity of the interfering fields. This is crucial when it comes to sensor movement and vibration in high static background fields like the inhomogeneous magnetization field of the component itself or the earth magnetic field. However, the measured voltage of a gradiometer depicts a gradient field which can make the interpretation of defect signals somewhat more complicated and absolute magnetic field values cannot be derived unequivocally afterwards. However, this does not affect the detectability of defects and in most cases the gradient character of the measured signal makes defect reconstruction possible.

**Figure 2.**
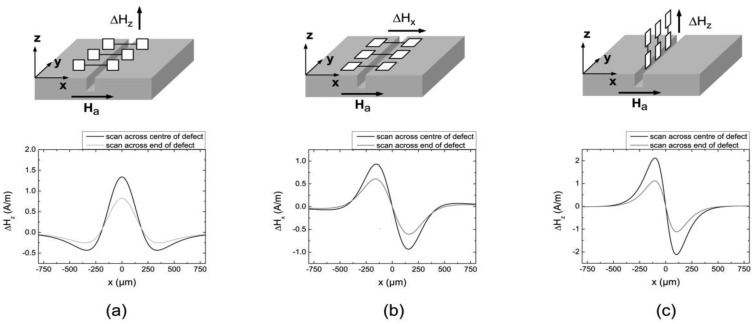
Scheme of different gradiometer alignments measuring the differences of one field component for different spatial directions (**a**) Δ*H_z_*(*x*_1_,*x*_2_) (**b**) Δ*H_x_*(*x*_1_,*x*_2_) and (**c**) Δ*H_z_*(*z*_1_,*z*_2_). Below the schemes simulated MFL signals are shown for each alignment. The black curves depict the signal when scanning across the centre of the crack in *x*-direction, grey when scanning across one of the edges of the defect.

There are several possible designs for gradiometric sensor arrangements. As shown in Figure 2(a), a tangential gradiometer may be used where the elements are aligned parallel to the surface of a test object, being sensitive to the normal field component. The measured signal is then proportional to Δ*H_z_*(*x*_1_,*x*_2_) = *H_z_*[area 1 (*x*_1_)] − *H_z_*[area 2 (*x*_2_)], *i.e.*, the difference of the normal magnetic field in tangential spatial direction. A significant limitation of this arrangement is the fact that it is not really suited to detect cracks in every direction without rotating the sensor. Assuming a defect parallel to the base line (not shown in Figure 2), a detection of this defect is hardly possible since both active layers have the same distance to the crack and measure the same field distribution. This leads to a zero gradient signal. A signal would only be detectable at the edges of a defect, which generally is insufficient for reliable crack detection. Keeping in mind that the magnetic field distributions get blurred with increasing lift off (*LO*), a too small base line BL will inevitably reduce the SNR due to similar field strengths observed by both active layers. In order to prevent this effect, the base line *BL* has to exceed the corresponding spatial signal width of a defect signal. On the other hand, a base line *BL* much larger than the spatial signal width leads to a separation of the detected MFL signal and could be misinterpreted. Finally, manufacturing problems both due to the thin film growth technology and the electrical connections are further limitations for this arrangement.

This is also true for the arrangement shown in Figure 2(b) where the surface normal of the sensor areas is perpendicular to the surface of the test specimen measuring the horizontal field component *H_x_*. This configuration experiences similar limitations as the previous arrangement. Most notably is the fact that cracks aligned parallel to the base line cannot be detected without rotating the sensor. Therefore, both arrangements (a) and (b) are not universally suitable for an automated NDT.

The above mentioned limitations can be overcome by using a normal gradiometer where the sensing elements are arranged perpendicular to the surface of the specimen, as shown in Figure 2(c). This gradiometer detects the difference of the normal field component Δ*H*_z_ normal to the surface, measuring the normal magnetic field *H_z_*. Considering the 1/*r*^2^-attenuation of the MFL-signal strength, the GMR layer close to the surface measures a significantly higher defect response compared to its counterpart positioned further away, leading to a magnetometer like response signal but also supporting the suppression of homogeneous noise sources. This type of sensor array is also capable of detecting defects in all directions without rotating the sensor and is not restricted by manufacturing considerations. For these reasons we chose the normal gradiometric arrangement (see Figure 2(c)) for our analysis presented in the following.

## 4. Normal Gradiometer

**Figure 3.**
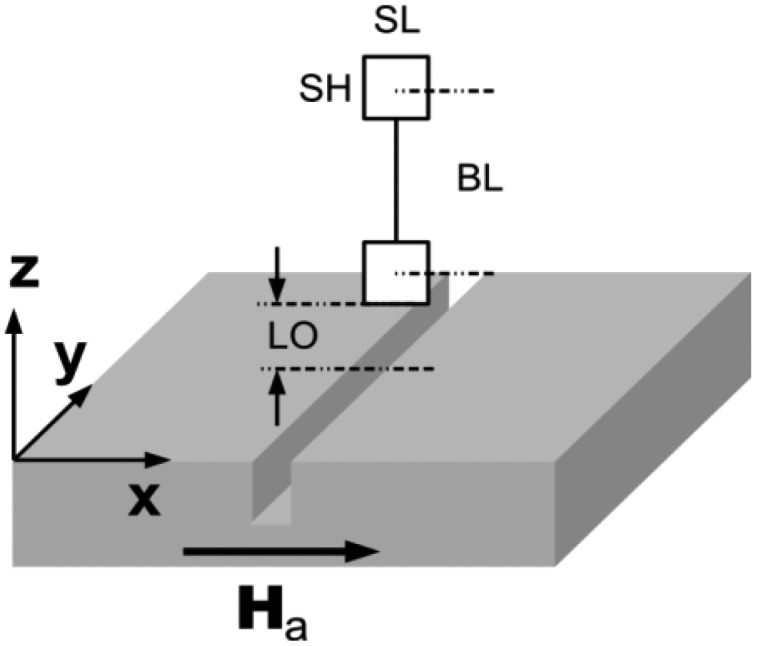
Parameters characterizing the dimensions of the gradiometer and its elements and the distance between sensor and surface (*LO* = lift off). The sensor area is *SH* × *SL*. The base line *BL* denotes the distance between the two centers of the active sensing layers.

Figure 3 shows the geometrical parameters of a gradiometer. These are the lift off *LO* describing the distance between the bottom edge of the lower active layer and the surface, the length *SL* and the height *SH* of the area of the active sensing layers, and the base line *BL* which denotes the distance between the two centres of the active layers of a gradiometer.

As a consequence of the gradient character of the MFL distinct changes of the magnetic field occur in the μm-regime. Therefore, the GMR sensor areas with a finite size of some μm^2^ detect a magnetic gradient field that varies over sensing area. This means that a different resistivity will be found across the sensor area. The total change of the resistance of the whole sensor is proportional to the integral of the gradient of the detected field component across the sensing area. In order to account for this gradient field in our parameter study, we calculated the mean value of the magnetic field component by integrating over the sensor area.

**Figure 4.**
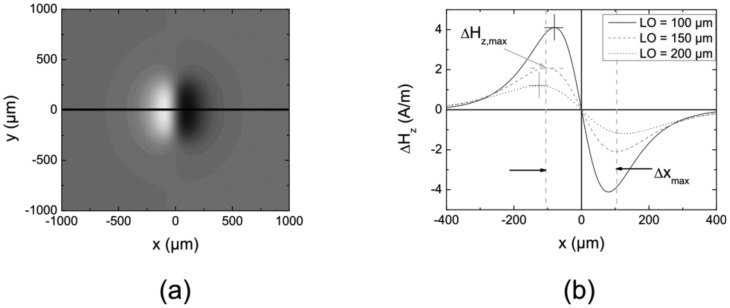
(**a**) Simulated MFL as grayscale representation for a crack with dimensions *t* = 50 μm, *w* = 2 μm and *l* = 500 μm; (**b**) Line scans across the centre of the crack (black line in (**a**)). For different *LO* the two parameters Δ*H_z_*_,max_ and Δ*x*_max_ characterize the signal. The crosses represent the *x*-position, at which Δ*H_z_* is maximum.

Figure 4(a) illustrates a greyscale picture of a simulated magnetic flux leakage for a crack with the dimensions *t* = 50 μm (depth), *w* = 2 μm (width) and *l* = 500 μm (length) for a *LO* of 150 μm. The magnetic permeability *μ_r_* was set to 1,000 and the applied field *H*_a_ inside the material was 100 A/m. The levels of grey correspond to a differential field Δ*H*_z_ of the normal field component of the MFL normal to the surface.

Line scans along the black solid line in Figure 4(a) are shown in Figure 4(b) for three different *LO* values. The maximum magnetic differential field amplitude Δ*H_z_*_,max_ and the distance between the extreme values Δ*x*_max_ are accentuated. It can be seen that with increasing *LO* the maximum field Δ*H_z_*_,max_ decreases and the distance Δ*x*_max_ increases, leading to somewhat more blurred signatures. These two parameters characterize the MFL signal and are the basis for the following parameter study.

### 4.1. Size Effects

**Figure 5.**
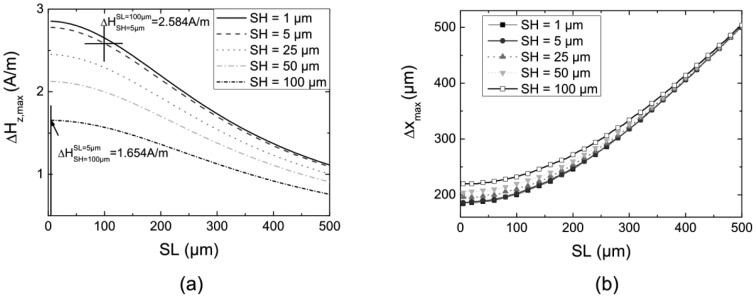
Simulation results of the normal gradiometer with *LO* = 150 μm and *BL* = 250 μm (defect: depth 50 μm, width 2 μm and length 500 μm). (**a**) Maximum MFL Δ*H*_z,max_ as function of the sensor length *SL* for different sensor heights *SH*. Two magnetic field points are highlighted for sensors with the same sensor area but with different *SL* and *SH*; (**b**) Diagram of the distance Δ*x*_max_ as a function of *SL* for several *SH*.

First, we investigate the influence of the size *SH* × *SL* (in principle, the GMR stripes are organized in a meander structure which form the sensor area *SH* × *SL*. The resistance of the whole active layer depends on the length of those stripes. Usually, the resistance of GMR sensors is in the order of some kΩ) of the active layers on the signal of the normal gradiometer. Figure 5(a,b) shows the calculated maximum signal amplitude Δ*H_z_*_,max_ and the distance Δ*x*_max_ as a function of the length *SL* of the sensor area, which were obtained from a line scan across the centre of a crack. The base line *BL* was 250 μm and the lift off *LO* was 150 μm. The multiple lines depict different heights *SH* of the area of the active layers.

Figure 5(a) shows Δ*H_z_*_,max_ as a function of the length *SL*. With increasing *SL* the area of the element increases, which in turn leads to an increasing distance between some parts of the active layers and the defect, especially if the defect is smaller than the length of the single GMR element. As a consequence these parts are penetrated by a lower magnetic field and, therefore, the mean values Δ*H*_max_ decrease. Similar results are observed for an increasing *SH*—the signal decreases with increasing *SH* due again to an averaging effect. Overall, the largest signal amplitude Δ*H*_max_ results from both minimal *SH* and *SL*. Comparing the influence of *SH* and *SL* for small values, it can be seen that the height has a stronger effect on Δ*H_z_*_,max_, e.g., for *SL* = 5 μm and *SH* = 100 μm the magnetic field gradient Δ*H_z_*_,max_ = 1.654 A/m is considerably smaller than for the same area with *SL* = 100 μm and *SH* = 5 μm (Δ*H_z_*_,max_ = 2.584 A/m).

Figure 5(b) shows Δ*x*_max_ as a function of *SL*. For *SL* > 300 μm the simulations show almost linear behavior. The reason is the integrating character of the sensor elements and the distance Δ*x*(*H*_max,min_–centre crack) between the extreme values of the MFL and the centre of the defect, which depends on the *LO*. If *SL* is larger than Δ*x*(*H*_max,min_–centre crack), the detected extreme values Δ*H*_max,min_ are found when one edge of the sensor is close to the centre of the defect. This leads to a separation of the measured positions of the extreme values when scanning across the defect which correlates with the length of the sensor, *i.e.*, Δ*x*_max_ ≈ *SL*. For small *SL*, thinking of a point-like measurement, the distance Δ*x*_max_ approaches a constant value, depending on the lift off *LO*. Smaller *LOs* lead to a smaller Δ*x*_max_, as shown in Figure 4(b). The demands of the testing problem and the technical capabilities, *i.e.*, the available distance between sensor and surface, confine the selection of the sensor geometry in terms of the required spatial resolution. Therefore, the length *SL* of the sensor should not exceed the sensor-to-surface distance. The same behavior can be observed for larger values of *SH* as shown in Figure 5(a). Here, the upper parts of the elements have a larger *LO*, resulting in a reduced field due to the 1/*r*^2^-dependence of the MFL.

Summarizing, the investigation of the influence of the size of the active GMR-layer shows that the best results can be obtained with a minimal, *i.e.*, ‘point-like’, sensor size. However, the resistance of ‘point-like’ GMR-elements is in the order of some ten Ω. The applied current should not exceed a few mA. To prevent damage under continuous operation the applied voltage has to be reduced. This leads to a decrease of the SNR since external noise is independent of the applied voltage and is not preferable for real industrial applications. In that case, increasing the area of the sensor in terms of a long and thin stripe structured like a meander is necessary to increase the resistance, hence leading to a better SNR. In conclusion, an increasing length *SL* has less influence on the signal size than an increasing height *SH*. For an adapted sensor design we therefore recommend to increase the length of the sensors to a reasonable size (*SL* = 100 − 200 μm, depending on the testing problem), while keeping the height at a minimum.

### 4.2. Influence of the Base Line

The base line *BL* of a gradiometer controls two major measuring quantities: the strength of the gradient signal and the external noise rejection. Generally, the baseline should be adapted to the spatial distribution or width of the crack signal, which in our case also corresponds to the defect width and the lift off. For *BL* larger than the spatial extension of the crack signal no loss of SNR due to spatial averaging will occur, since for the normal configuration the gradiometer then works like a magnetometer. However, noise due to external background fields becomes more relevant. For *BL* distinctly smaller than the extension of the crack signal the measured magnetic field strength will be nearly the same at both active areas of the gradiometer. Thus, the signal strength decreases, even if background clutter is also suppressed more efficiently. As a result an optimum *BL* has to be found.

**Figure 6.**
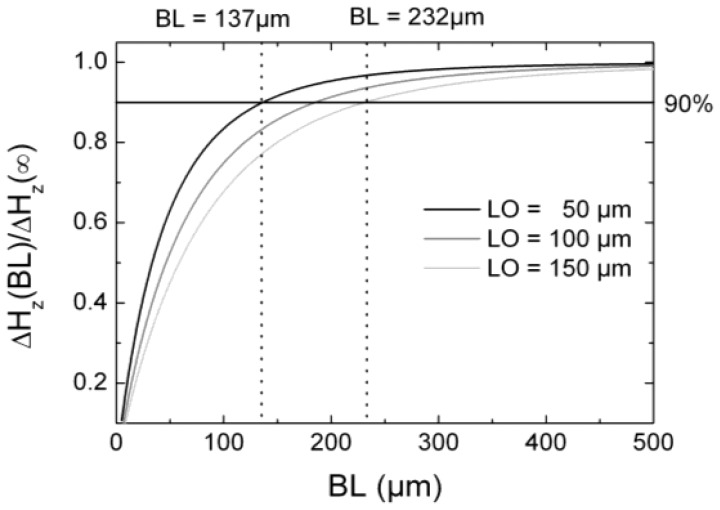
Normalized maximum MFLs as a function of the base line *BL* for a sensor with dimensions *SL* × *SH* = 100 × 5 μm^2^, and for different *LOs*. The straight line depicts the case for a gradient field of 90% of the magnetometer signal. Depending on the *LO*s, this 90% response is obtained for base lines *BL* = 137 μm (*LO* = 50 μm) and *BL* = 232 μm (*LO* = 150 μm).

In Figure 6 the normalized differential field Δ*H_z_*_,max_(*BL*)/ Δ*H_z_*_,max_(∞) is presented as a function of the base line *BL*. As active layer area we chose *SL* × *SH* = 100 × 5 μm^2^, a reasonable value obtained from Figure 5, and a *LO* of 50 μm, 100 μm, and 150 μm, respectively. We observe that a *BL* < 50 μm leads to a small difference between the measured fields of both areas of the gradiometer as mentioned above and, therefore, to a small signal. A *BL* ≥ 500 μm shows an approximately constant value, *i.e.*, approaching a magnetometer-like signal (Δ*H_z_*_,max_(∞) = *H_z_*_,max_(magnetometer)), since only the sensor layer near the surface is efficiently penetrated by the magnetic stray flux. On the other hand, we already mentioned that the external noise and slightly shifting background fields become more significant for larger *BL*. In Figure 6 the dashed lines show the *BL* values for which the gradiometer signals are 90% of the magnetometer signals for a *LO* of 50 μm (*BL* = 137 μm) and a *LO* of 150 μm (*BL* = 232 μm). Taking into consideration that the suppression of far-field inhomogeneous background fields generally scales proportional to *BL*, background clutter should be remarkably suppressed for base lines around 200 μm, even if it stems from the tested component itself. In conclusion, for realistic lift offs below 150 μm a gradiometer with a base line of 250 μm should be an adequate choice for a NDT-adapted sensor, since the gradiometric signal is in the order of 90% of a magnetometer and the background fields should be sufficiently reduced.

### 4.3. Layout of the GMR Array

So far only the optimization of single sensors have been investigated. However, for industrial applications measuring time and cost play a key role in most cases. This industrial need may be satisfied by the application of a sensor array instead of a single sensor. In the following we present an investigation of the effects that have to be considered when GMR sensor elements are arranged in a sensor array. These sensor arrays offer better performance in terms of measuring time and cost, and the amplitudes of each single element signal can be used for the estimation of the defect size. Nevertheless, scanning with an array across a small defect might lead, depending on the position of the defect, to sensor signals with different amplitudes. Here, the size of the sensor elements compared to the dimensions of the defect as well as the distance between sensor element and defect play an important role. In the following, we therefore investigate two cases in more detail.

First, a sensor array is analyzed, where the plane of the active layers is perpendicular to the direction of the defect. We assume that one element of the sensor array scans exactly with its centre along a defect, *i.e.*, in *y*-direction, with one half of the active layer on the left side and one half on the right. In this case the element above the defect does not detect a field gradient, *i.e.*, the detected mean value of the MFL is zero. Therefore, the adjacent elements have to detect a sufficient MFL signal to gather enough information for a decision whether a specimen has a defect or not.

**Figure 7.**
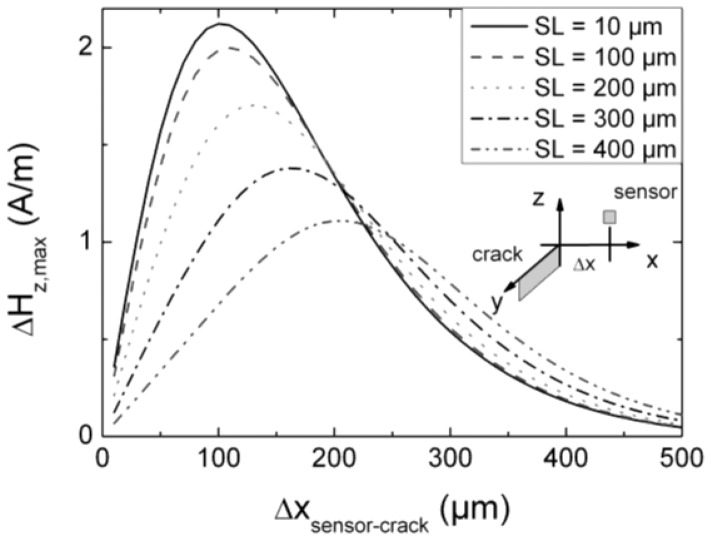
Simulations for the case that the surface normal of the sensor array is aligned parallel to the defect (see scheme inside of the diagram). The scanning direction is along the defect in *y*-direction. The diagram shows the maximum field difference Δ*H_z,max_* of a sensor element as a function of the distance Δ*x* between sensor and defect for several *SL*. The baseline *BL* was 250 μm, *LO* = 150 μm and *SH* = 50 μm.

In this consideration we define the distance between the centre of the active layers of adjacent elements and the centre of the defect as Δ*x*_sensor-crack_ (scheme in Figure 7), which is larger than *SL*. In Figure 7 the signal Δ*H_z_*_,max_ is shown as a function of Δ*x*_sensor-crack_ for different *SL* (*LO* = 150 μm, *BL* = 250 μm, *SH* = 50 μm). The position of the maximum field depends on *SL*, as shown in Figure 5(b). For a sensor with *SL* = 400 μm the maximum field value Δ*H_z_*_,max_ = 1.11 A/m is found at Δ*x* = 200 μm. However, in our assumption the position of an adjacent element for a sensor array with *SL* = 400 μm is, at best, Δ*x* = 400 μm, which corresponds to a mean value of the maximum magnetic field Δ*H_z_*_,max_ ≤ 0.34 A/m. In this case the amplitude of the magnetic field is reduced by more than 300%! Less dramatic reductions are observed for *SL* = 300 μm and *SL* = 200 μm. Only for sensor lengths *SL* ≤ 100 μm signals close to the maximum are possible. From that example we learn that in order to be able to reliably detect defects centered right below one sensor in a sensor array, the length of each sensor should not exceed a maximum value. This maximum value depends on the shape of the signal, mainly Δ*x*, which again depends mainly on the width of the defect and the lift off. In our case of small defects and small lift offs, *SL* should not exceed 200 μm.

**Figure 8.**
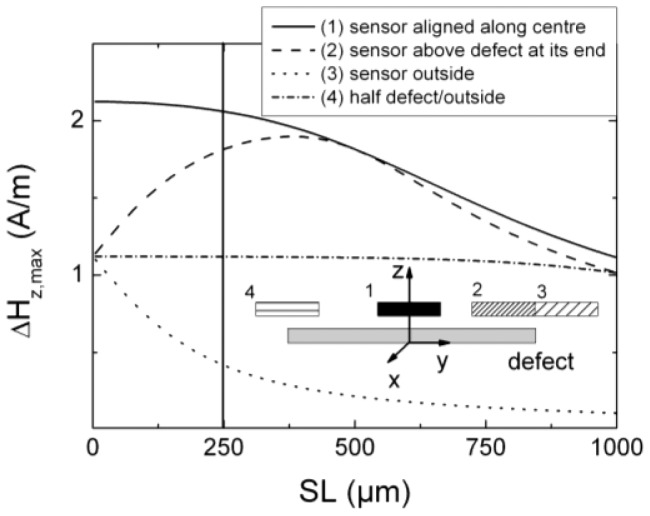
Sensor scanning across the crack with a length of 500 μm along *x*-direction (*BL* = 250 μm, *LO* = 150 μm and *SH* = 50 μm). The sensor is aligned parallel to the defect. The scheme shows the different sensor positions above the defect, labelled 1, 2, 3, and 4. Details are described in the text.

In the 2nd case the sensor elements are aligned parallel to the defect with the scanning direction perpendicular to it, *i.e.*, along the *x*-direction, and one element crosses the defect above its centre (see scheme of the black sensor in Figure 8). If the distance between two adjacent elements or the elements themselves are too large detailed information might be lost which is necessary for a precise reconstruction of the defect geometry, especially the length.

In Figure 8 we show Δ*H_z_*_,max_ as a function of *SL* for different positions with respect to the defect. The black curve represents the maximum value of the defect signature when scanning along the *x*-direction across the centre of the defect (case 1 depicted in Figure 8). The grey dashed line represents the field values for crossing the defect at one of its edges with the whole sensor length being located above the defect (case 2 in Figure 8). The light grey dotted line shows the behavior if the sensor element is arranged at the edge but outside of the defect (case 3 in Figure 8).

For case 1 (black curve in Figure 8) the maximum Δ*H_z_*_,max_ decreases monotonously with increasing sensor length *SL*. Towards the edges the MFL signal decreases and less magnetic field spreads in the surrounding. The mean value of the detected field is diminished. In case 2, a sensor scanning across the edge of the defect (dashed line in Figure 8), the length of the active layers increases along the defect length. At *SL* = 500 μm case 1 and 2 show the same amplitude, since both alignments are now identical and scanning exactly across the defect centre. With increasing *SL* the signal decreases stronger than for case 1 due to the larger distance between one edge of the defect and the end of the sensor. The dotted line (case 3 in Figure 8) depicts the behavior if the sensor scans outside of the defect with one edge of the sensor close to the defect. Here, the mean value signal decreases significantly with increasing distance due to less measurable MFL signals far away from the defect. In case 4 (the dotted-dashed line in Figure 8) the value is approximately constant before it starts to decrease for large values of *SL*.

Thus, for an appropriate sensor array with sufficiently high spatial resolution the sensor length should not exceed half of the smallest defect dimension which has to be detected. If the length of the sensor exceeds this limit the spatial resolution is reduced and determination of the defect length is hardly possible. For a sensor with *SL* = 250 μm (perpendicular line in Figure 8) and a defect length of 500 μm two cases should be considered. First, two elements cross the defect and detect the same MFL signal of Δ*H_z_*_,max_ = 1.82 A/m as derived from Figure 8. The neighboring elements outside of the defect then measure a MFL of Δ*H_z_*_,max_ = 0.42 A/m. This represents a reduction of more than a factor of 4. The two centre elements should therefore detect the defect with a SNR of at least 8 in order to be able to also detect the defect with the adjacent elements as further redundant information. Secondly, one element could cross the defect above its centre. Then the field value is Δ*H_z_*_,max_ = 2.06 A/m in the example configuration. The neighboring elements then scan only partially across the defect with one half above the defect and one half outside. From this it follows that the measured mean value of the MFL decreases slightly, *i.e.*, these elements measure a maximum magnetic field of Δ*H_z_*_,max_ = 1.12 A/m. Nevertheless, in this case the information gained from the three sensor elements is sufficient for a defect evaluation concerning the defect length.

## 5. Simulation *vs*. Measurement

In order to verify some of the results of the simulations we carried out measurements of the normal magnetic field component *H_z_* of the MFL of a defined defect for different *LO* values. An artificial defect with a depth of 110 μm, a length of 5,500 μm and an opening of 90 μm (smaller defects are hardly possible) was introduced in a steel plate by low energy EDM (electrical discharge machining). We chose especially low erosion rates to maintain the magnetic properties in the vicinity of the notch. Since the fabrication of GMR gradiometers with different base lines *BL* is rather complex, a magnetometer (GF792, Sensitec GmbH, Lahnau, Germany) with a sensing area of 20 × 17 μm^2^ was used. We therefore carried out measurements after magnetizing the steel plate at different lift offs and calculated the gradient field Δ*H_z_* (*BL* = *LO*1 − *LO*2) = *H_z_* (*LO*1) − *H_z_* (*LO*2). These differential fields for a fixed sensor area were compared with simulations concerning the base line *BL* of a gradiometer (see Section 4.2).

**Figure 9.**
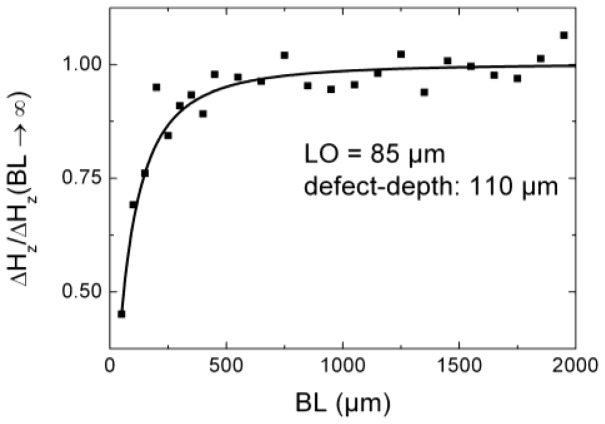
Normalized gradiometer signal as a function of the base line *BL*. The solid line depicts the simulation and the squared dots the measurement results. The EDM defect has a length of 5,500 μm, a depth of 110 μm and an opening of 90 μm. The *LO* was 85 μm.

In Figure 9 we compare simulation (line) and experiment (dots) of the normalized field Δ*H_z_*/Δ*H_z_*(*BL* = 50 μm) as a function of the base line *BL*. Using the normalized field gives a signal that is independent of the internal applied field and the permeability of the material, *i.e.*, the knowledge of the material parameters is not necessary since these quantities are eliminated in the normalized equation of *H_z_*. The distance between the nearest sensor position and the surface was 85 μm, hence defining the *LO* of a gradiometer with different base lines. Except for *BL* = 200 μm, the relative errors are below 6%.

In our simulations we included the area-dependency of the detected MFL signals, *i.e.*, the field gradient due to the spatial extension of the stray field of a defect. The field values at different positions along the active sensing area are different due to inhomogeneities of the stray field. The measured signals of a GMR sensor are mean values depending on the size of the sensing area of the active layers. Comparing the experimental results and the simulations we found a good agreement, as shown in Figure 9. Hence, we assume that the simulations are also well suited for GMR signals as a GMR active area homogeneously contributes to the total field value in terms of a superposition and linear weighted field contributions along the sensing area.

## 6. Conclusions/Outlook

In this paper we carried out a sensor parameter study for an optimal design of GMR sensor arrays adapted for NDT. Our investigation is based on an analytical model [15] describing the MFL-distribution of simple rectangular defects.

We investigated a normal gradiometer, since this configuration leads to a noise reduction and all defect orientations can be detected with one single sensor without additional rotation during testing. The design study included the variation of the sensor parameters, *i.e.*, independently the length *SL* and height *SH* of the active layers, and the base line *BL* (the distance between two active layers in a gradiometric configuration). We found that small active layers give the best results both in terms of absolute signal strength and spatial resolution. Hence, active layers with an area of less than 10 μm^2^ are preferable if a high spatial resolution is required and small defects have to be detected. However, in industrial applications measuring time plays an important role, demanding a compromise between spatial resolution and measuring time. Hence the smallest defect length to be detected should be used as guideline for the limitation of the sensor parameters. For example, if the smallest defect to be detected has a length of 500 μm, a sensor length of 200 μm is a reasonable value to achieve a certain degree of redundancy, *i.e.*, detection of the defect by more than one sensor. It is important, though, to keep the height of the sensor area at a minimum, since an increase of the height strongly reduces the amplitude of the mean MFL signal. A reduction of the base line reduces the external noise, but also affects the amplitude of the gradiometric MFL signals. For small defects, as it was the case in this investigation, a baseline *BL* = 250 μm leads to a reduction of the amplitude of around 8% compared to a magnetometer and seems to be a good compromise. Using a GMR magnetometer, we investigated the influence of the baseline on the signal amplitude experimentally. We found a good agreement between simulation and experiment, hence being able to demonstrate the reliability of the analytical equations in terms of simulating GMR sensor layers.

Using the results of this parameter study the next step will be a design of several NDT-adapted GMR sensor arrays aiming for automated testing. Especially for simple geometries, arrays with 16 or more elements are preferable to reduce the amount of measuring time. Also, a wafer, once it is designed, provides a high cost-effectiveness. Finally, employing GMR-technology in magnetic testing has the potential to bridge the gap between mm-sized induction coils and the scanning magnetic force microscope detecting the magnetic field distribution on the nm-scale.

The results are also helpful for optimizing GMR sensor layouts in alternative application cases when detecting gradients of magnetic fields in the μm-range. This could be measuring of positions, lengths, angles or electrical currents. Even if the field sources are somewhat different the findings of this NDT-GMR can be very helpful.
